# Supplementary figures and images for: The expression and clinical significance of serine hydroxymethyltransferase2 in gastric cancer
Source: PeerJ. 2024 Jan 4;12:e16594. doi: 10.7717/peerj.16594 (PMC10771762; doi:10.7717/peerj.16594)

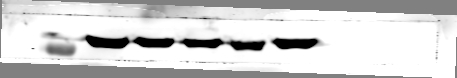

Supplement: Supplemental Information 1 [file peerj-12-16594-s001.zip › peerj-82521-original_data/original data/Actin.jpg]

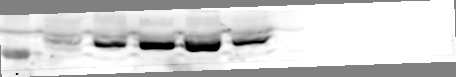

Supplement: Supplemental Information 1 [file peerj-12-16594-s001.zip › peerj-82521-original_data/original data/SHMT2.jpg]
